# Development and validation of hybrid Brillouin-Raman spectroscopy for non-contact assessment of mechano-chemical properties of urine proteins as biomarkers of kidney diseases

**DOI:** 10.1186/s12882-020-01890-x

**Published:** 2020-06-15

**Authors:** Abduzhappar Gaipov, Zhandos Utegulov, Rostislav Bukasov, Duman Turebekov, Pavel Tarlykov, Zhannur Markhametova, Zhangatay Nurekeyev, Zhanar Kunushpayeva, Alisher Sultangaziyev

**Affiliations:** 1 Department of Clinical Sciences, Nazarbayev University School of Medicine, Nur-Sultan, Kazakhstan 010000; 2 Department of Physics, School of Sciences and Humanities, Nazarbayev University, Nur-Sultan, Kazakhstan 010000; 3 Department of Chemistry, School of Sciences and Humanities, Nazarbayev University, Nur-Sultan, Kazakhstan 010000; 4 Department of Internal Medicine, Astana Medical University, Nur-Sultan, Kazakhstan 010000; 5 Department of Proteomics and Mass Spectrometry, National Center for Biotechnology, Nur-Sultan, Kazakhstan 010000

**Keywords:** Brillouin light scattering, Raman scattering, SERS, Urine proteomics, Non-contact diagnostics, Proteinuria, Viscoelastic, Chemical, Biochemical

## Abstract

**Background:**

Proteinuria is a major marker of chronic kidney disease (CKD) progression and the predictor of cardiovascular mortality. The rapid development of renal failure is expected in those patients who have higher level of proteinuria however, some patients may have slow decline of renal function despite lower level of urinary protein excretion. The different mechanical (visco-elastic) and chemical properties, as well as the proteome profiles of urinary proteins might explain their tubular toxicity mechanism. Brillouin light scattering (BLS) and surface enhanced Raman scattering (SERS) spectroscopies are non-contact, laser optical-based techniques providing visco-elastic and chemical property information of probed human biofluids. We proposed to study and compare these properties of urinary proteins using BLS and SERS spectroscopies in nephrotic patient and validate hybrid BLS-SERS spectroscopy in diagnostic of urinary proteins as well as their profiling. The project ultimately aims for the development of an optical spectroscopic sensor for rapid, non-contact monitoring of urine samples from patients in clinical settings.

**Methods:**

BLS and SERS spectroscopies will be used for non-contact assessment of urinary proteins in proteinuric patients and healthy subjects and will be cross-validated by Liquid Chromatography-Mass Spectrometry (LC-MS). Participants will be followed-up during the 1 year and all adverse events such as exacerbation of proteinuria, progression of CKD, complications of nephrotic syndrome, disease relapse rate and inefficacy of treatment regimen will be registered referencing incident dates. Associations between urinary protein profiles (obtained from BLS and SERS as well as LC-MS) and adverse outcomes will be evaluated to identify most unfavored protein profiles.

**Discussion:**

This prospective study is focused on the development of non-contact hybrid BLS - SERS sensing tool and its clinical deployment for diagnosis and prognosis of proteinuria. We will identify the most important types of urine proteins based on their visco-elasticity, amino-acid profile and molecular weight responsible for the most severe cases of proteinuria and progressive renal function decline. We will aim for the developed hybrid BLS - SERS sensor, as a new diagnostic & prognostic tool, to be transferred to other biomedical applications.

**Trial registration:**

The trial has been approved by ClinicalTrials.gov (Trial registration ID NCT04311684). The date of registration was March 17, 2020.

## Background

Urine generally represents the fluid biopsy of the kidney, providing an alternative to blood plasma as a potential source of disease biomarkers [1, 2]. Therefore, discharged urine can serve as the human biofluid medium for noninvasive assessment of the kidney health status [3]. Healthy urine contains many end-product metabolites, electrolytes and few amounts of small proteins (selective proteins, that filtered from glomerular baseline membrane), that called albumin in normal range of up to 20–30 mg/dL. Any excessive range of albumin more than 30 mg/dL or appearance “big proteins” (non-selective proteins) called microalbuminuria or proteinuria [4].

Proteinuria is recognized as a major marker of chronic kidney disease (CKD) severity, the predictor of renal function decline and cardiovascular mortality [5–8]. The main pathway of progressive renal scarring is caused by tubular toxicity of filtering proteins, so more, the rapid development of end-stage kidney disease (ESKD) is expected in those patients who have higher level of proteinuria [9, 10]. However, despite high level of proteinuria, paradoxically, some patients may have slow decline of renal function compared to those patients with lower level of urinary protein excretion. This may possibly due to their different mechanical (viscosity, elasticity and compressibility) and chemical properties, as well as the different proteomic structure of the excreting urinary proteins [11]. Thus, there is a continuing uncertainty, why severity of renal failure progression could differ between proteinuric patients?

To date, different diagnostic methods in clinical settings can be used to ascertain urinary proteins. The extent of proteinuria can be gauged using an albumin-specific dipstick, immunochemical techniques, and high-performance liquid chromatography [12, 13]. However, results may vary between the methods, possibly due to different type, origin and size of excreting urinary proteins. The quality and quantity of filtered proteins, especially their mechanical properties such as physical viscosity [14, 15] compressibility [16] and chemical properties [17], such as amino-acid sequence (type of peptides), and molecular weight (low, middle and high molecular weight proteins), identify what kind of urinary proteins are more toxic and hazardous [1, 17]. Further studies including different areas of biomedicine and biophysics are needed to better understand importance of mechano-chemical characteristics of urinary proteins, in providing diagnostic and prognostic information that influences patient care and outcomes.

Measuring the viscosity, elasticity and chemistry of biological fluids is an important analytical tool in diagnostic, prognostic, and preventive medicine and research [14, 15]. The viscosity of biological solvents helps in gaining insight into the kinetics and dynamics of molecular and cellular processes such as conformational changes in protein [18, 19]. Such optical techniques as Brillouin and Raman spectroscopies to analyze human biofluids are very attractive for potential non-contact and fast clinical diagnostics with minimal or no sample preparation, without alteration of the fluid obtained from the body, representing a powerful simplification over most current techniques. Recently, dual Brillouin-Raman spectroscopy has been demonstrated as a viable diagnostic sensing tool in variety of biomedical applications [20–23].

The goal of the project is to develop and validate combined Brillouin & Surface-Enhanced Raman Scattering (SERS) Spectroscopy technique for simultaneous non-contact assessment of visco-elastic and chemical properties of human urine proteins as biomarkers of kidney disease. Systematic studies of these properties in proteins of urine samples to be taken from diseased and healthy subjects will be cross-validated by Liquid Chromatography Mass Spectrometry (LC-MS). The project ultimately aims for development of optical spectroscopic sensor for rapid, non-contact monitoring of urine samples from patients in clinical setting.

## Methods

### Study design

**Fig. 1:**
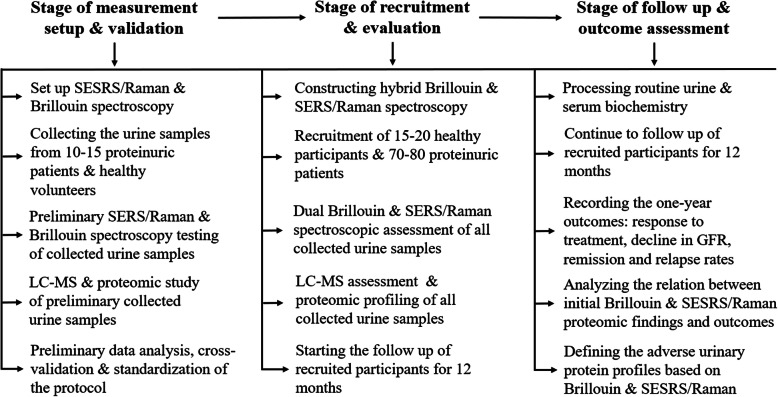
Flow diagram illustrating study design, tasks and procedures. SERS: surface enhanced Raman scattering, LC-MS: Liquid Chromatography-Mass Spectrometry, GFR: glomerular filtration rate

This project is prospective observational validation study, including subsequently the following three stages: measurement validation and protocol development (10–14 months), participant recruitment and evaluation (4–6 months), follow up and outcome assessment (10–14 months). Each stage consists of defined tasks, presented in Fig. 1.

### Participants

The 80 patients with proteinuria will be recruited form National Scientific Medical Center, admitted to the internal medicine department. Age and gender matched 20 healthy volunteers will be enrolled from the outpatient clinics. The inclusion criteria are both male and female patients with any ethnic group, aged between 18 and 65 years old and newly diagnosed patients with glomerulonephritis and different range of proteinuria. The following exclusion criteria will be used: patients aged < 18 and > 65 years, glomerular filtration rate (GFR) < 60 ml/min, pregnant females, patients with diabetes mellitus, cancer, infectious diseases, and other life-treating comorbidities/conditions. Clinical and demographics data of the participants, medical history, comorbidities and data related to the disease will be recorded in case report forms.

### BLS spectroscopy

**Fig. 2:**
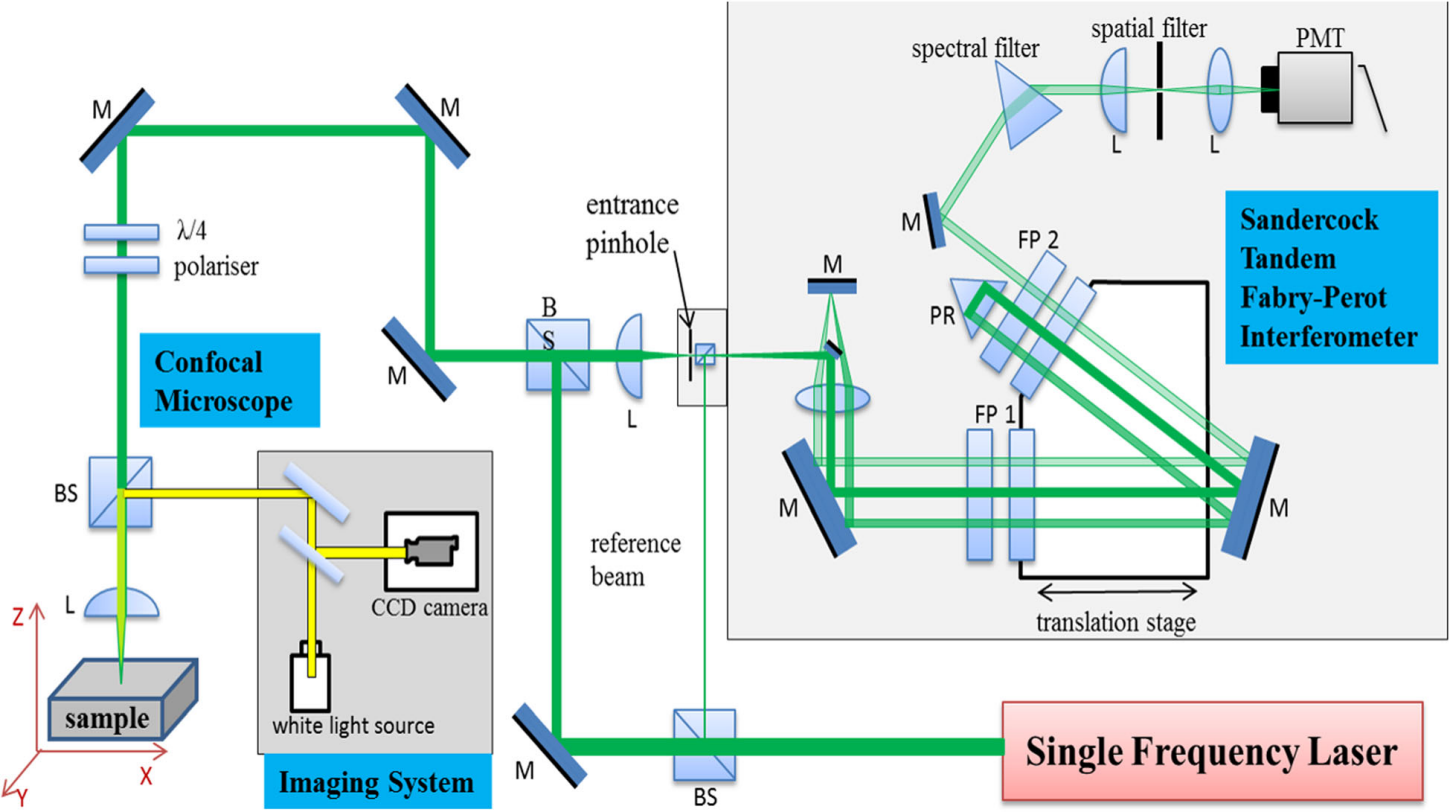
Schematic demonstration of BLS Spectroscopy using scanning 6-pass TFPI coupled with confocal microscope. M: Mirror, F: Filter, L: Lens or Objective, IL: Imaging Lens, BS: Beam Splitter, CCD: Charge-Coupled Device, TFPI: tandem Fabry-Perot interferometer, PR: prism, PMT: photo-multiplying tube

BLS spectroscopy (Fig. 2) will be performed using Torus single longitudinal mode 532 nm laser (Laser Quantum, England) in conjunction with high contrast scanning 6-pass tandem Fabry-Perot interferometer (TFPI) coupled with confocal microscope and imaging system (JRS Instruments, Switzerland) and separately by high speed Brillouin hyperfine spectrometer employing virtual image phase array (VIPA) (Light Machinery, Canada) to measure Brillouin spectra from human biofluids for assessment of their viscoelastic properties.

### SERS/Raman spectroscopy

**Fig. 3:**
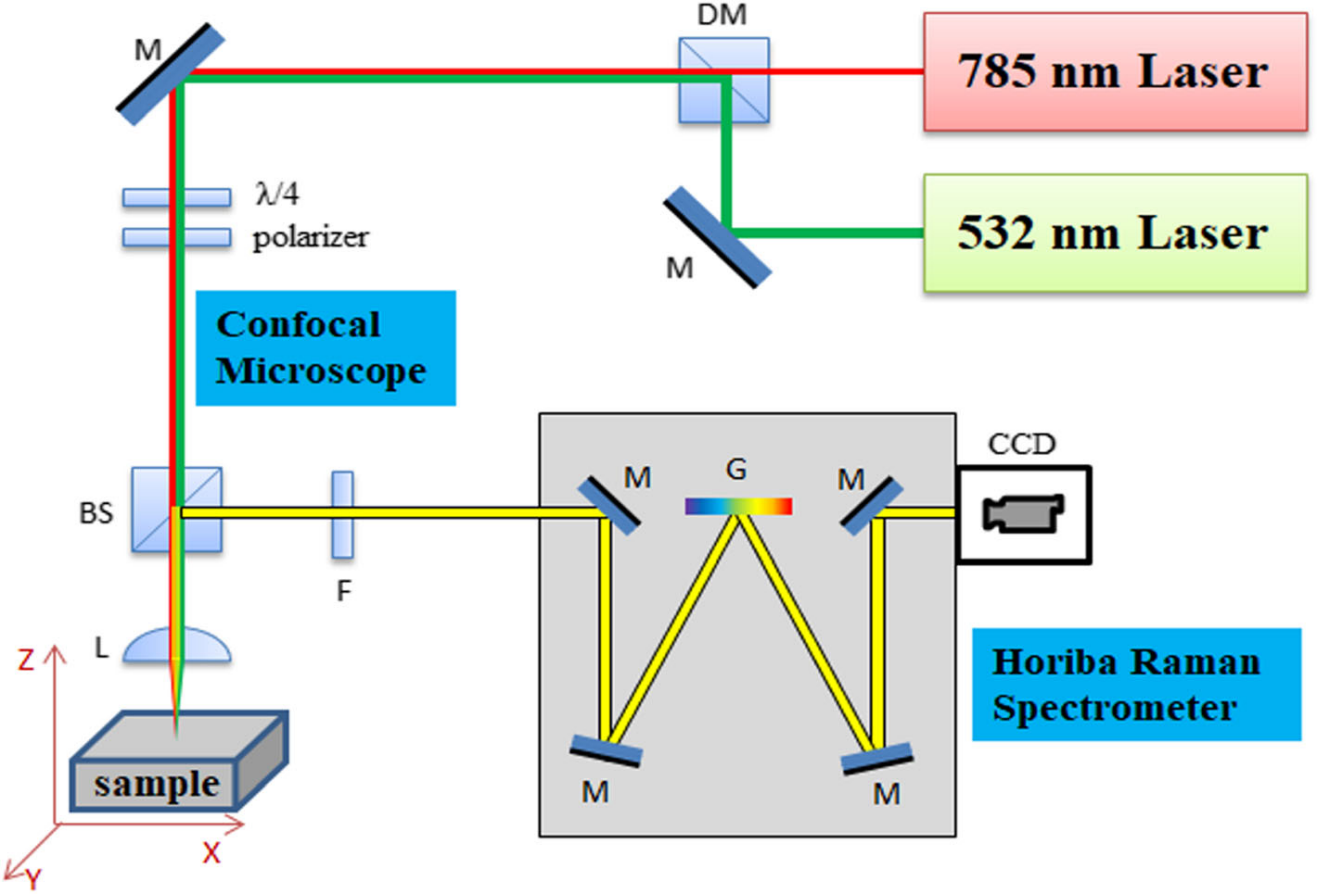
Schematic demonstration of Raman Spectroscopy. M: Mirror, F: Filter, L: Lens or Objective, BS: Beam Splitter, DM: Dichroic Mirror, G: Grating, CCD: Charge-Coupled Device

Raman spectroscopy (Fig. 3) will perform using Horiba LabRAM HR Raman microscope with 785 nm laser excitation and × 100 or × 10 microscope objectives. The SERS spectroscopy will be performed on urine samples stirred with commercial 60 nm diameter gold nanoparticles (742,015, from Sigma Aldrich, UK). Prior to mixing with urine samples, those nanoparticles would go through three centrifugation/ resuspension cycles in order to remove stabilizing agents/reagents from the nanoparticle suspension as described in publication of Gudun et al., [24]. After stirring with urine, taken from patients, suspension of gold nanoparticles will be dropcasted on some spots on commercial gold film coated slides (CA134 from Dynasil Corp., USA), they will get dried and multiple Raman spectra will be measured on those spots for each sample with above mentioned Raman microscope and those spectra will be averaged.

### Hybrid Brillouin-SERS system

**Fig. 4:**
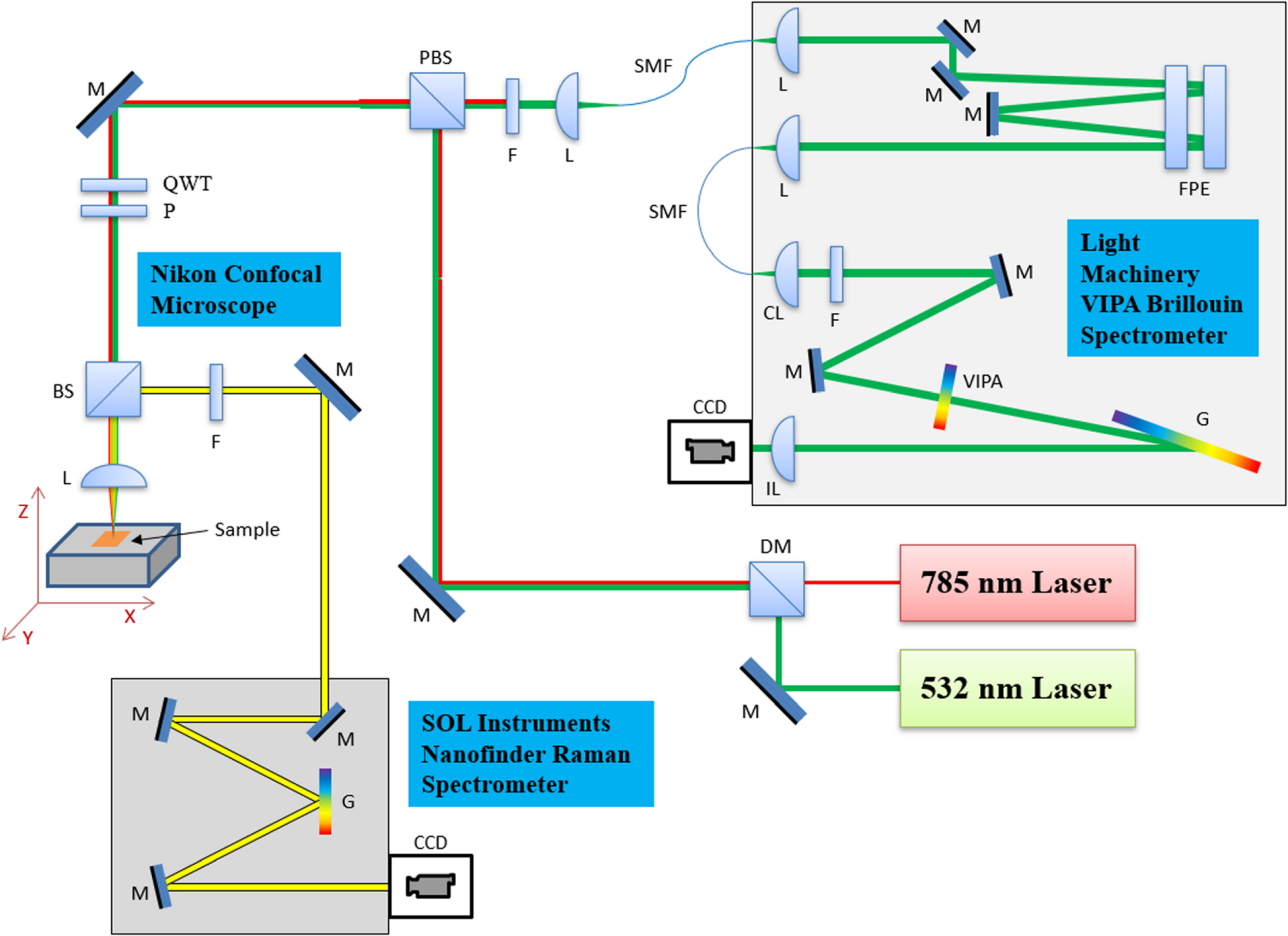
Schematic demonstration of hybrid BLS-Raman Micro-Spectroscopy sensing system. M: Mirror, F: Filter, L: Lens or Objective, CL: Cylindrical Lens, IL: Imaging Lens, BS: Beam Splitter, PBS: Polarizing Beam Splitter, DM: Dichroic Mirror, SMF: Single Mode Fiber, G: Grating, FPE: Fabry-Perot Etalon, CCD: Charge-Coupled Device, VIPA: virtual image phase array, QWT: quarter-wave plate

We propose to construct hybrid Brillouin-Raman system, which will simultaneously assess both visco-elastic (i.e. mechanical) and chemical properties at the same time from the same excitation laser source (532 nm or 780 nm). In this arrangement our Brillouin hyperfine spectrometer (Light Machinnery, Canada) system will be coupled with confocal microscope (Nicon, Japan) and Nanofinder Raman spectrometer (SOL Instruments, Belarus) and motorized sample stage (Standa, Lithuania) for 3D imaging The schematic of the proposed hybrid all-optical system is displayed in the Fig. 4.

### Liquid chromatography-mass spectrometry

A reversed-phase LC-MS will perform for profiling of urine protein. LC-MS system consists of a Dionex HPLC pump (Ultimate 3000 RSLCnano System, Thermo Scientific) and UHR-QqTOF mass spectrometer Impact II (Bruker). Protein sample will be injected through trapping column setup Acclaim PepMap100 C18 5 mm × 300 um pre-column (Thermo Scientific) and peptides separated on an analytical Acclaim Pep-Map 15 cm x 75um RSLC column (Thermo Scientific) using 75 min multistep acetonitrile gradient (buffer A: 0.1% FA; buffer B 90%ACN/ 10% H2Oin 0.1% FA) at a flow rate of 0.3 ul/min. Data analysis will be performed through Mascot software using the SwissProt protein database. Furthermore, protein samples differentiated according to their molecular weights will be separately assessed by dual Raman-Brillouin hybrid system for comparative mechano-chemical characteristics and correlations.

### Blood and urine samples

Full blood biochemistry and corresponding urinalysis will provide additional information about participant health condition, such as functional condition of liver, kidney, heart, metabolic status etc. These widely evaluated routine laboratory data will be obtained from all participants. Routine blood metabolic profile including total protein, albumin, renal and liver function tests, coagulation tests, lipid profiles as well as urinalysis, proteinuria, albumin to creatinine ratio (ACR) and protein to creatinine ratio (PCR) will be performed at the study enrollment. Control renal function tests and ACR or PCR will be performed at the end of the follow up period. All blood and urine samples from participants will be collected at the time of hospital admission or at the visits to outpatient clinic. All blood and urine samples will be stored at -80co freezers for proper preservation before further visco-elastic and chemical analysis will be performed. After last patient is enrolled to the study, all laboratory evaluations will be provided according to the study protocol.

### Ethics statement

The project team is strongly committed to comply with the ethical policy. We guarantee that information provided in the frame of this project is reliable and unique. We confirm our adherence to research ethics, particularly preventing any fabrication and falsification of research data resulting in distorted research data, plagiarism and fake authorship. All work within the project will be conducted in accordance with international and national legal and ethical principles. All participants will follow ethical, health, safety and other relevant regulations. Laboratory research team and service contractors will be blinded from patients’ clinical data to assure clinically non-biased research. The knowledge acquired by this project will be completely disseminated via conventional academic channels such as international and national conference presentations and scientific publications. In order to defend intellectual property, the results of the project will be published in reputable peer-reviewed scientific journals of international standing.

### Outcome measures

The primary outcome of the study is to develop and validate the Brillouin and SERS/Raman spectroscopy for non-contact urinary protein detection and protein profiling in patients with nephrotic syndrome. The secondary outcome of the study is identifying the most hazarded protein profiles associated with adverse outcomes.

### Statistical analysis

All statistical analyses will be performed using STATA MP Version 15 (STATA Corporation, College Station, TX) statistical package. Two-sided *P-*values less than 0.05 will be reported as a significant for all analyses. Mean ± standard deviation (SD) for normal and median - interquartile range (IQR) for abnormal distribution of numerical variables will be shown in all data. Categorical variables will be presented as numbers (percentage). Categorical variables will be compared using Chi-square test or Fisher’s exact test and continuous variables will be compared using Kruskal–Wallis and/or Mann–Whitney U-test, as appropriate. Comparison of repeated two or more variables will be assessed with Wilcoxon and/or Friedman test as appropriate.

Obtained measurements of urinary protein by Brillouin and SERS/Raman spectroscopy will be compared with LC-MS data from same participant urine samples. Correlation between Brillouin and SERS/Raman spectroscopy parameters, LC-MS data and clinical-laboratory data of patients as well as healthy participants will be provided using Pearson’s or Spearman’s correlation as appropriate.

Univariate logistic regression will be used to test the association of several risk factors with high level of proteinuria. Multivariate logistic regression analysis will be used to assess the predictors for adverse outcomes. A receiver operator characteristics curve (ROC curve) analysis will be performed to identify the sensitivity, specificity, negative and positive predictive values of cut-off levels of Brillouin and SERS/Raman spectroscopy parameters in prediction of high proteinuria. Between measurement comparison of AUC will be provided with method developed by DeLong et al. [25].

During 1 year of follow-up, all adverse events such as exacerbation of proteinuria, progression of CKD, complications of nephrotic syndrome (thrombosis, nephrotic crisis, infection, including death), disease relapse rate and inefficacy of treatment regimen will be registered with referencing incident dates. Unadjusted and adjusted regression models will be evaluated to identify the association between urinary protein profiles defined by Brillouin and SERS/Raman spectroscopy and abovementioned adverse outcomes.

## Discussion

To the best of our knowledge, there has been no prior investigation of human urine fluids to diagnose proteinuria using by neither Brillouin nor Raman spectroscopy, nor by SERS.

Recently, dual Brillouin-Raman spectroscopy has been demonstrated as a viable diagnostic sensing tool in variety of biomedical applications [20–23]. Brillouin spectroscopy has been traditionally used in material science [26]. Measured Brillouin frequency peak position for a material is given by ${\omega}_B=\frac{2 nV}{\lambda}\cos \frac{\theta }{2}$ where *n* is refractive index, *λ* is incident laser wavelength, *θ* is laser light scattering angle and *V* is a sound velocity in the probed fluid medium. Knowing *V* and density of medium *ρ*, one can evaluate longitudinal elastic bulk modulus by *E* = *ρV*^2^. From measured Brillouin peak’s full width at half maximum (FWHM) *∆ω*, one can determine the total viscosity of the fluid ${\eta}_{total}=\frac{{\Delta  \omega}_B{\lambda}^2\rho }{8{\pi}^2{n}^2}$. Lately Brillouin spectroscopy has been experiencing a renaissance, especially, with respect to non-invasive diagnostics of bulk biomaterials [27–33], including works done in our Brillouin spectroscopy laboratory [30–34]. Brillouin spectroscopy offers direct way to assess phase transformations of proteins [34] and a possible method of quantifying total protein concentration. As the protein concentration of a solution changes, so does the elasticity of the fluid, expressed as bulk modulus of compressibility [16, 29].

On the other hand, Raman spectroscopy is able to provide information about chemical structure, phase and polymorphic state, crystallinity and molecular interactions of biologic samples, including urine proteins [35–37]. It should be noted that, although water, urea, creatinine and uric acid are detectable by using conventional Raman scattering spectroscopy, the detection of Albumin protein in urine is very challenging due to low detection sensitivity of this technique to albumin concentration in urine [35] even in proteinuric samples. Therefore, there have been attempts to boost Raman signal by means of SERS spectroscopy [38, 39], as well as our efforts to boost Brillouin light scattering signal using surface plasmon enhancement [40–43]. Each method of this study will give a valuable knowledge and full contribution to the aim and task of our study. Brillouin spectroscopy offers a possible method of quantifying total protein concentration without alteration of the fluid obtained from the body, representing a powerful simplification over most current techniques. As the protein concentration of a solution changes, so does the elasticity of the fluid, expressed as bulk modulus of compressibility. Raman spectroscopy provides detailed information about chemical structure, phase and polymorphy, crystallinity and molecular interactions of any biologic samples, including urine proteins.

LC-MS is able to detect all types of urinary protein (which could be missed by nephelometry immunoassay method) and sequencing the amino-acid properties as well as grades the proteins according to their molecular weight (e.g. low, middle and high molecular weight proteins). Previous studies demonstrated that LC-MS could identify low-weight molecular urinary proteins that cannot be detectable in routine immunochemistry assay, and early discover microalbuminuria in diabetes and hypertension [44, 45]. However, application of LC-MS in daily clinical practice is less reliable due to its cost and sophisticated procedures. Thus, the application of contactless optical evaluation methods such as Brillouin and Raman spectroscopies, to determine the mechano-chemical properties of urinary proteins and its cross-validation with LC-MS would provide scientific and clinical interest.

Summarizing the available relevant literature and emphasizing the scientific novelty and significance of dual Brillouin-SERS spectroscopy, we will try to study and cover following question in this proposed project: Do non-contact dual Brillouin-SERS spectroscopy provide differential and diagnostic information on mechano-chemical profile of urine and urinary protein in healthy subjects and patients with different range of proteinuria?

### Expected science & technology, social & economic impacts

In case of successful development of non-contact hybrid Brillouin-SERS sensing tool and its clinical deployment for diagnosis and prognosis of proteinuria, we expect its application for diagnosis and prognosis of other renal diseases of patients with abnormalities related not just to high protein loss, but also to elevated concentration of urea, creatinine, uric acid and other components. The impact of to-be-developed optical instrumentation and knowledge base is also envisioned in other areas of biomedical sciences and medicine, including biophotonic sensing of viruses, where non-invasive optical diagnostics and imaging is currently in high demand.

Young scientists in Kazakhstan will be trained in important multidisciplinary areas spanning from nephrology to biomedical photonics & engineering, laser-optical instrumentation design and construction, spectroscopy, biomechanics, biorheology and biochemistry of human fluids. By the end of 3-year project we expect:

1. to develop basic science foundation for measurement and analysis of visco-elastic and chemical properties of human urine and urine proteins of patients having various degree of proteinuria.
2. to construct, optimize and test the hybrid Raman-Brillouin spectroscopy sensing system for non-contact simultaneous monitoring of visco-elastic and chemical properties of urine samples taken from healthy subjects and patients with different range of proteinuria and other kidney diseases related to elevated concentration of urea, creatinine and uric acid and cross-validate them with LC-MS.
3. to identify the most important types of urine proteins based on their visco-elasticity, amino-acid profile and molecular weight responsible for the most severe cases of proteinuria and progressive renal function decline.
4. to reach a roadmap for translating hybrid Brillouin-Raman sensor as a new diagnostic & prognostic tool in patients with proteinuria to a clinical setting and its potential deployment to other biomedical applications.
5. to report research results in at least 3 international conferences and publish at least 5 papers in peer-reviewed international journals with high impact factors.
6. to train young Kazakh medical and physical scientist in advanced inter-disciplinary areas of nephrology & biophotonics.

## Data Availability

The datasets produced after the completion of this trial may be requested from the principal investigator (AG).

## Abbreviations

CKD: Chronic kidney disease
BLS: Brillouin light scattering
SERS: Surface enhanced Raman scattering
LC-MS: Liquid Chromatography-Mass Spectrometry
ESKD: End-stage kidney disease
GFR: Glomerular filtration rate
VIPA: Virtual image phase array
ACR: Albumin to creatinine ratio
PCR: Protein to creatinine ratio
TFPI: Tandem Fabry-Perot interferometer

## References

[1] Harpole M, Davis J, Espina V (2016). Current state of the art for enhancing urine biomarker discovery. Expert Rev Proteomics.

[2] Gaipov A, Taubaldiyeva Z, Askarov M, Turebekov Z, Kozina L, Myngbay A (2019). Infusion of autologous bone marrow derived mononuclear stem cells potentially reduces urinary markers in diabetic nephropathy. J Nephrol.

[3] Brunzel NA (2013). Fundamentals of urine and body fluid analysis-E-book: Elsevier health sciences.

[4] D'Amico G, Bazzi C (2003). Pathophysiology of proteinuria. Kidney Int.

[5] Culleton BF, Larson MG, Parfrey PS, Kannel WB, Levy D (2000). Proteinuria as a risk factor for cardiovascular disease and mortality in older people: a prospective study. Am J Med.

[6] Oh SW, Baek SH, Kim YC, Goo HS, Heo NJ, Na KY (2012). Mild decrease in estimated glomerular filtration rate and proteinuria are associated with all-cause and cardiovascular mortality in the general population. Nephrol Dialysis Transplant.

[7] Solak Y, Yilmaz MI, Siriopol D, Saglam M, Unal HU, Yaman H (2015). Serum neutrophil gelatinase-associated lipocalin is associated with cardiovascular events in patients with chronic kidney disease. Int Urol Nephrol.

[8] Solak Y, Yilmaz MI, Saglam M, Demirbas S, Verim S, Unal HU (2013). Mean corpuscular volume is associated with endothelial dysfunction and predicts composite cardiovascular events in patients with chronic kidney disease. Nephrology..

[9] Baines RJ, Brunskill NJ (2011). Tubular toxicity of proteinuria. Nat Rev Nephrol.

[10] Bruzzi I, Benigni A, Remuzzi G (1997). Role of increased glomerular protein traffic in the progression of renal failure. Kidney Int Suppl.

[11] Zhou H, Yuen PS, Pisitkun T, Gonzales PA, Yasuda H, Dear JW (2006). Collection, storage, preservation, and normalization of human urinary exosomes for biomarker discovery. Kidney Int.

[12] Viswanathan G, Upadhyay A (2011). Assessment of proteinuria. Adv Chronic Kidney Dis.

[13] Alkhamaisah SI, Younes KM, Gaipov A, Aljofan M. Development and validation of a simple and sensitive HPLC method for the determination of liquid form of therapeutic substances. Electron J Gen Med. 2019;16(6):1-8.

[14] Srivastava N, Davenport RD, Burns MA (2005). Nanoliter viscometer for analyzing blood plasma and other liquid samples. Anal Chem.

[15] Fainerman VB, Trukhin DV, Zinkovych II, Miller R (2018). Interfacial tensiometry and dilational surface visco-elasticity of biological liquids in medicine. Adv Colloid Interf Sci.

[16] Wang S, Lee L, Lee J (2001). A linear relation between the compressibility and density of blood. J Acoust Soc Am.

[17] Julian BA, Suzuki H, Suzuki Y, Tomino Y, Spasovski G, Novak J (2009). Sources of urinary proteins and their analysis by urinary proteomics for the detection of biomarkers of disease. Proteomics Clin Appl.

[18] Ansari A, Jones CM, Henry ER, Hofrichter J, Eaton WA (1992). The role of solvent viscosity in the dynamics of protein conformational changes. Science..

[19] Keith AD, Snipes W (1974). Viscosity of cellular protoplasm. Science..

[20] Mattana S, Mattarelli M, Urbanelli L, Sagini K, Emiliani C, Serra MD (2018). Non-contact mechanical and chemical analysis of single living cells by microspectroscopic techniques. Light Sci Appl.

[21] Scarponi F, Mattana S, Corezzi S, Caponi S, Comez L, Sassi P (2017). High-performance versatile setup for simultaneous Brillouin-Raman microspectroscopy. Physical Review X.

[22] Meng Z, Bustamante Lopez SC, Meissner KE, Yakovlev VV (2016). Subcellular measurements of mechanical and chemical properties using dual Raman-Brillouin microspectroscopy. J Biophotonics.

[23] Traverso AJ, Thompson JV, Steelman ZA, Meng Z, Scully MO, Yakovlev VV (2015). Dual Raman-Brillouin microscope for chemical and mechanical characterization and imaging. Anal Chem.

[24] Gudun K, Elemessova Z, Khamkhash L, Ralchenko E, Bukasov R. Commercial gold nanoparticles on untreated aluminum foil: versatile, sensitive, and cost-effective SERS substrate. J Nanomater. 2017; 2017:1-8.

[25] DeLong ER, DeLong DM, Clarke-Pearson DL (1988). Comparing the areas under two or more correlated receiver operating characteristic curves: a nonparametric approach. Biometrics..

[26] Dil J (1982). Brillouin scattering in condensed matter. Rep Prog Phys.

[27] Scarcelli G, Yun SH (2007). Confocal Brillouin microscopy for three-dimensional mechanical imaging. Nat Photonics.

[28] Beroud J, Vincent B, Paternotte E, Nguyen VS, Kerdjoudj H, Velot E (2013). Brillouin spectroscopy: a new tool to decipher viscoelastic properties of biological scaffold functionalized with nanoscale films. Biomed Mater Eng.

[29] Steelman Z, Meng Z, Traverso AJ, Yakovlev VV (2015). Brillouin spectroscopy as a new method of screening for increased CSF total protein during bacterial meningitis. J Biophotonics.

[30] Akilbekova D, Ogay V, Yakupov T, Sarsenova M, Umbayev B, Nurakhmetov A (2018). Brillouin spectroscopy and radiography for assessment of viscoelastic and regenerative properties of mammalian bones. J Biomed Opt.

[31] Akilbekova D, Yakupov T, Ogay V, Umbayev B, Yakovlev VV, Utegulov ZN. Brillouin light scattering spectroscopy for tissue engineering application. Optical Elastography and Tissue Biomechanics V. Vol. 10496. Proceedings of SPIE. 2018. 104961I. San Francisco, United States. 10.1117/12.2289923.

[32] Coker Z, Troyanova-Wood M, Traverso AJ, Yakupov T, Utegulov ZN, Yakovlev VV (2018). Assessing performance of modern Brillouin spectrometers. Opt Express.

[33] Rakymzhan A, Yakupov T, Yelemessova Z, Bukasov R, Yakovlev VV, Utegulov ZN. Monitoring of vegetation drying by Brillouin and Raman spectroscopies. Sensing for Agriculture and Food Quality and Safety IX. Vol. 10217. Proceedings of SPIE. 2017. 102170C. Anaheim, United States. 10.1117/12.2263535.

[34] Dmitriev A, Vashchenkov V, Fedoseev A, Lushnikov S (2019). Phase transformations of bovine serum albumin: evidences from Rayleigh-Brillouin light scattering. J Raman Spectrosc.

[35] Premasiri WR, Clarke RH, Womble ME (2001). Urine analysis by laser Raman spectroscopy. Lasers Surg Med.

[36] Brindha E, Rajasekaran R, Aruna P, Koteeswaran D, Ganesan S (2017). High wavenumber Raman spectroscopy in the characterization of urinary metabolites of normal subjects, oral premalignant and malignant patients. Spectrochim Acta A Mol Biomol Spectrosc.

[37] Rygula A, Majzner K, Marzec KM, Kaczor A, Pilarczyk M, Baranska M (2013). Raman spectroscopy of proteins: a review. J Raman Spectrosc.

[38] Zou Y, Huang M, Wang K, Song B, Wang Y, Chen J (2016). Urine surface-enhanced Raman spectroscopy for non-invasive diabetic detection based on a portable Raman spectrometer. Laser Phys Lett.

[39] Chamuah N, Saikia A, Joseph AM, Nath P (2019). Blu-ray DVD as SERS substrate for reliable detection of albumin, creatinine and urea in urine. Sensors Actuators B Chem.

[40] Meng Z, Yakovlev VV, Utegulov Z. Surface-enhanced Brillouin scattering in a vicinity of plasmonic gold nanostructures. Plasmonics in Biology and Medicine XII. Proceedings of SPIE. 2015. 93400Z-1. San Francisco, United States. 10.1117/12.2079667.

[41] Johnson WL, Kim SA, Utegulov Z, Shaw J, Draine BT (2009). Optimization of arrays of gold nanodisks for plasmon-mediated Brillouin light scattering. J Phys Chem C.

[42] Johnson W, Kim S, Utegulov Z, Draine BT. Surface-plasmon fields in two-dimensional arrays of gold nanodisks. Plasmonics: Metallic Nanostructures and Their Optical Properties VI; Proceedings of SPIE. 2008;7032:70321S-1.

[43] Utegulov Z, Shaw JM, Draine BT, Kim S, Johnson WL, editors. Surface-plasmon enhancement of Brillouin light scattering from gold-nanodisk arrays on glass. Plasmonics: Metallic Nanostructures and Their Optical Properties V; Proceedings of SPIE. 2007;6641:66411M.

[44] Wang Z, Hoy WE, Nicol JL, Wang Z, Su Q, Atkins RC (2008). Predictive value of nephelometric and high-performance liquid chromatography assays of urine albumin for mortality in a high-risk Aboriginal population. Am J Kidney Dis.

[45] Polkinghorne KR, Su Q, Chadban SJ, Shaw JE, Zimmet PZ, Atkins RC (2006). Population prevalence of albuminuria in the Australian diabetes, obesity, and lifestyle (AusDiab) study: immunonephelometry compared with high-performance liquid chromatography. Am J Kidney Dis.

